# Temporal Prediction of Paralytic Shellfish Toxins in the Mussel *Mytilus galloprovincialis* Using a LSTM Neural Network Model from Environmental Data

**DOI:** 10.3390/toxins14010051

**Published:** 2022-01-12

**Authors:** Jisun Shin, Soo Mee Kim

**Affiliations:** 1 BK21 School of Earth and Environmental Systems, Pusan National University, Busan 46241, Korea; sjs1008@pusan.ac.kr; 2 Maritime ICT R&D Center, Korea Institute of Ocean Science and Technology (KIOST), Busan 49111, Korea; 3 Department of Convergence Study on the Ocean Science and Technology, Korea Maritime and Ocean University, Busan 49111, Korea

**Keywords:** paralytic shellfish toxins, *Mytilus galloprovincialis*, LSTM neural network model

## Abstract

Paralytic shellfish toxins (PSTs) are produced mainly by *Alexandrium catenella* (formerly *A. tamarense*). Since 2000, the National Institute of Fisheries Science (NIFS) has been providing information on PST outbreaks in Korean coastal waters at one- or two-week intervals. However, a daily forecast is essential for immediate responses to PST outbreaks. This study aimed to predict the outbreak timing of PSTs in the mussel *Mytilus galloprovincialis* in Jinhae Bay and along the Geoje coast in the southern coast of the Korea Peninsula. We used a long-short-term memory (LSTM) neural network model for temporal prediction of PST outbreaks from environmental data, such as water temperature (WT), tidal height, and salinity, measured at the Geojedo, Gadeokdo, and Masan tidal stations from 2006 to 2020. We found that PST outbreaks is gradually accelerated during the three years from 2018 to 2020. Because the in-situ environmental measurements had many missing data throughout the time span, we applied LSTM for gap-filling of the environmental measurements. We trained and tested the LSTM models with different combinations of environmental factors and the ground truth timing data of PST outbreaks for 5479 days as input and output. The LSTM model trained from only WT had the highest accuracy (0.9) and lowest false-alarm rate. The LSTM-based temporal prediction model may be useful as a monitoring system of PSP outbreaks in the coastal waters of southern Korean.

## 1. Introduction

*Alexandrium* species belong to one of the major genera with respect to the diversity, magnitude, and consequences of harmful algal blooms (HABs) [1,2]. The more than 30 morphologically defined species in genus *Alexandrium* produce paralytic shellfish toxins (PSTs) [3]. It amasses in filter feeding bivalves, causing disease and death in human consumers, namely paralytic shellfish poisoning (PSP). Toxic dinoflagellates pose several problems to the aquaculture industry and continue to endanger human health [4,5,6,7].

In the case of Korea, spring blooms of *Alexandrium* species causing PSP are mainly affected by *Alexandrium catenella*. The blooms are distributed limitedly in the southeast coast, especially in Masan-Jinhae Bay and Geoje coasts [8,9]. In 1986 and 1996, the deaths of four people in Geoje and Busan were related to PSTs ingested through the consumption of the mussel *M. galloprovincialis* (formerly *M. edulis*) [10]. *M. galloprovincialis* is the most preferred shellfish in Korea after oysters. Although many peoples consumed mussels, they paid little attention to the potential for toxin contamination before the first accident in PSP in 1986 [8]. Since then, the PSP has emerged as a possible intimidation to public health and a primary trouble for the shellfish aquaculture industry. The second occurrence of PSP on the Geoje coast in relation to the consumption of mussels off the coast of Busan and Masan-Jinhae Bay in 1996 prompted the Korean government to pay more attention to the public action plan. To restrain harvesting and marketing of shellfish whose PST concentration exceeds the quarantine limit (80 μg STX diHCI equivalents 100 g^−1^), monitoring of shellfish toxicity is carried out in shellfish production area. Since 2000, the NIFS has provided information on PST-inducing species and PSP outbreaks every 1–2 weeks by default [11]. Additionally, such information may be provided at shorter intervals during an intensive period of PST outbreaks. However, regular data on at least a daily basis are be needed for an immediate response to PST outbreaks. In fact, if information pertaining to the *Alexandrium* blooms that produce PSTs could be obtained, PST outbreaks could be anticipated directly. Unfortunately, the red-tide breaking news provided by the NIFS does not often provide information about *Alexandrium* blooms, making it difficult to obtain information about such blooms.

Because the annual trend of PST outbreak is related to *A. catenella* blooms, it is essential to understand the factors controlling the development, maintenance, and decline of these blooms. Although physical and biological couplings, such as stratification and vertical migration, have been accepted as important mechanisms for *Alexandrium* bloom formation [12], it is difficult to generalize the environmental controls of bloom dynamics due to the complex interrelations between physical, chemical, and biological factors. Water temperature (WT) and salinity play essential role in cyst germination and cell growth of *A. catenella*. Baek et al. [13] investigated the environmental factors affecting the dynamics of *A. catenella* that generate PSTs along the Geoje coast during bloom season. PSTs were constantly found at levels above safe level for human consumption at 15 °C. The authors also reported that the amount of discharges from the Nakdong River affected the environmental conditions along the Geoje coast, which resulted in lower salinity and higher nutrient levels, promoting *A. catenella* blooms. *A. catenella* strain were grown within a wide WT range [14], but WT was optimum at 15 °C. In addition, *A. catenella* cells appeared when WT was from 10 to 19 °C, which means that WT range suitable for *A. catenella* growth. Kim et al. [15] reported that *A. catenella* populations swelled considerably from March to May, decreased sharply in June when WT passed 20 °C, and then reappeared in the winter season. Seasonal blooms of *A. catenella* occur mostly on the southern coast of the Korean Peninsula in spring [15,16]. These studies showed that the increase in *A. catenella* population can be affected by WT, especially since the optimum WT range in early spring and late autumn is relatively narrow, from 12 °C to 15 °C. Meanwhile, horizontal movement due to tidal effect affects the movement of *A. catenella* along the coast of Geojedo. In both 2017 and 2018, Back et al. [13] reported that the first PST outbreaks occurred off the coast of Busan coast near the Nakdong River. Then, PST-producing *A. catenella* populations developed in Jinhae Bay and at the Geoje coast during spring. They demonstrated that *A. catenella* blooms along the coast of Geoje may have been fortified by tidal currents. These findings help us qualitatively understand the environmental factors affecting the occurrence of PST-causing blooms but that there is a limitation for predicting the emergence of PSTs, which is important information for reducing losses caused by PSTs.

The objective of this study was to predict the timing of PST outbreaks in the mussel *M. galloprovincialis*. We used environmental data from three tidal stations and a long-short-term memory (LSTM) neural network model. The LSTM, a recurrent neural network, was used for time series predictions of environmental factors, such as red-tide blooms [17] and water quality [18]. First, we collected environmental measurements at three tidal stations and PST outbreak timing information provided by the NIFS from 2006 to 2020. Then, to fill in the missing data within the daily measured environmental data, we trained the LSTM regression model. Finally, an LSTM classification model was trained and evaluated for temporal prediction of PST outbreaks from environmental data.

## 2. Results

### 2.1. Periodic Tendency of Paralytic Shellfish Toxins Outbreak

**Figure 1:**
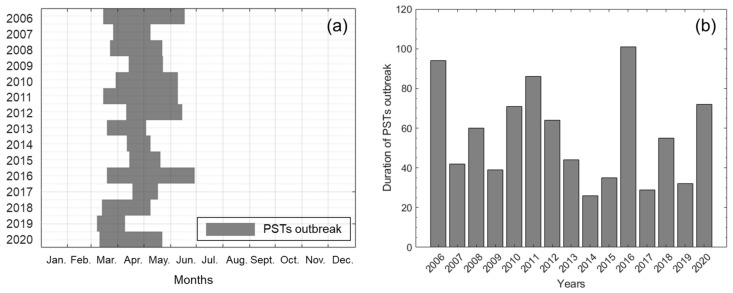
(**a**) Period and (**b**) duration of paralytic shellfish toxins (PSTs) outbreak from 2006 to 2020. The information was obtained by National Institute of Fisheries Science (NIFS).

Figure 1 shows the period and duration of PST outbreaks in the study area for the 15 years from 2006 and 2020. In general, PST outbreaks begin to occur around March and disappear around June (Figure 1a). In 2019, PSTs occurred at the earliest date of 7 March and disappeared by 8 April. On the other hand, in 2017, PSTs first occurred on 17 April, which is the latest date during the study period. We confirmed that the timing of PST outbreaks gradually accelerated during 2018–2020. As shown in Figure 1b, PSTs occurred for 850 days of the 5479 days. This number of days represents 16% of the total study period, and events occurred for an average of 57 days per year. In 2016, PSTs occurred for 101 days, which was the longest duration; in 2014, they occurred for 26 days, the shortest duration of days. The duration showed no specific tendency.

### 2.2. Gap-Filling of Environmental Factors

**Figure 2:**
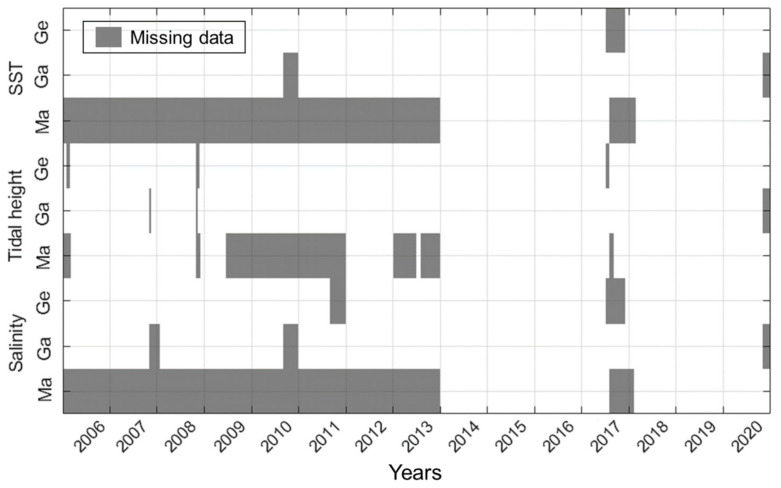
Missing patterns of environmental data for three tidal stations. Ge, Ga, and Ma correspond to Geojedo, Gadeokdo, and Masan tidal stations, respectively. Gray box represents the missing days of measurement.

To predict the timing of PST outbreaks, we used WT, tidal height, and salinity data obtained every day from tidal stations at Geojedo, Gadeokdo, and Masan from 2006 to 2020 (5479 days). Figure 2 shows the missing days among the environmental data. The Masan and Geojedo tidal stations had the highest and lowest missing data rates, respectively. The WT at the Masan station had the highest missing data rate of 57.1% (3127 days). Specifically, there were no data for the seven consecutive years (2922 days) from 2006 to 2013. The tidal height data at the Geojedo station had the lowest missing data rate of 1.8% (96 days). The three stations had no missing data in common from 1 January 2014 to 31 June 2017 (period 1) and from 1 February 2018 to 31 October 2020 (period 2). To fill the gaps of missing data days, we trained an LSTM regression model with the data measured during period 1 (1277 days) and tested the model with the period 2 data (1004 days).

Table 1 shows the environmental data gap-filling performance of the trained LSTM regression models. Among the three stations, we used measurements from only one station or the other two stations. The missed environmental factors of each station shown on the left of Table 1 were estimated with the measurements from the stations specified at the top of Table 1. In the case of two stations, the remaining station was estimated using the two other stations. The root mean squared errors (RMSEs) of WT estimation ranged from 0.84 to 2.22. The result showed the best performance when Geojedo data were gap-filled with Gadeokdo data. It showed the lowest performance when Masan data were gap-filled with Geoje data. The WT of the Geojedo and Masan stations had the lowest RMSE when estimating using Gadeokdo data, whereas Gadeokdo had the best results when using two stations. In the case of tidal height, the RMSEs ranged from 10.86 to 35.76. Unlike WT, the poor performance showed when estimating Geojedo data with Gadeokdo data. The lowest RMSEs for all stations showed when using 2 stations. The RMSEs of salinity were from 1.32 to 3.69. Among the station data, the Geojedo data showed the best performance (1.32–1.83), whereas the Masan data had the highest RMSEs (3.44–3.69). Unlike other environmental factors, the salinity at all three stations showed the best results when estimating the variable with only one station. To fill the day gaps of the missing environmental data, we chose the available LSTM regression model at each missing section. Among the three possible models for each station, the model with the smallest RMSE was selected first. If it could not be selected due to a lack of the factors needed to construct the model, a suboptimal model was selected.

**Figure 3:**
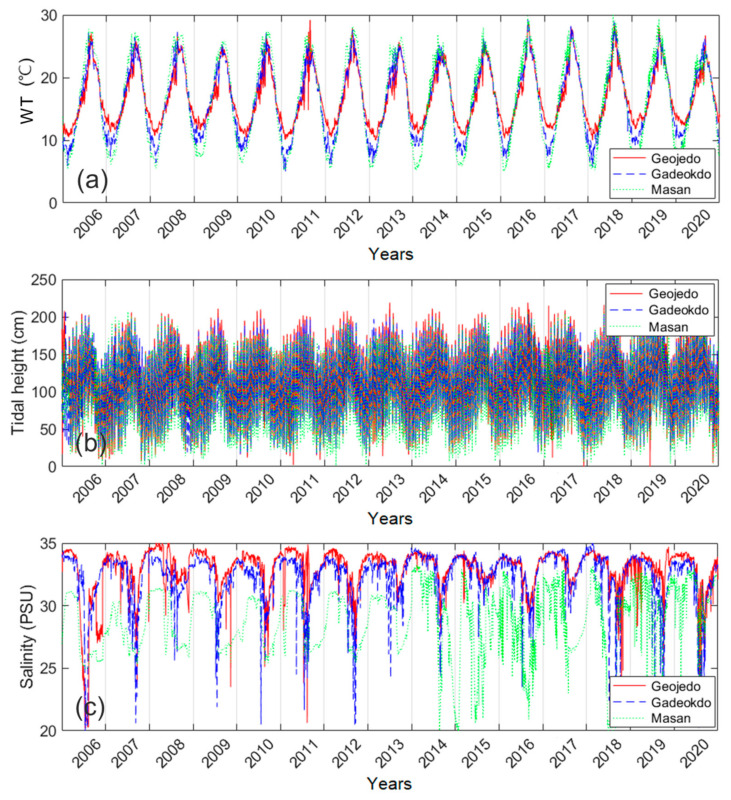
Temporal variations of the gap-filled environmental factors, (**a**) WT, (**b**) tidal height, and (**c**) salinity from 2006 to 2020. The red, the dashed blue, and the dotted green lines represent factors of Geojedo, Gadeokdo, and Masan stations, respectively.

We filled the day gaps of the daily environmental data (WT, tidal height, and salinity as oceanographic factors) to generate daily full-sequence data by LSTM regression models. Figure 3 shows the daily tendency of the environmental data from 2006 to 2020 after gap-filling. In the case of WT, the Masan station showed the largest variation (Figure 3a). The minimum and maximum WT values were 5 °C and 30 °C, respectively. On the other hand, the Geojedo station showed the smallest variation (10–29.2 °C). WT values gradually increased from March to August of each year. We calculated the R^2^ value to confirm the similarity between the gap-filled environmental factors of the three stations. The R^2^ of WT ranged from 0.87 to 0.96, showing high similarity. There was no clear difference in tidal height levels among the three stations (Figure 3b). The Geojedo station had the highest maximum tidal height level (243 cm), whereas the Gadeokdo station had the lowest maximum level (215 cm). The R^2^ of tidal height between stations was high (0.87–0.89). The Geojedo and Gadeokdo stations had similar patterns of salinity variation (Figure 3c). The salinity level of the Masan station with the lowest maximum level (33.3 PSU) was lower than that of other stations. The R^2^ of salinity between stations was the low (0.18–0.47).

The WT showed a dominant seasonal pattern every year, while tidal height fluctuated due to the influence of tidal currents irrespective of the season. Salinity was mainly dependent on the season. However, the salinity level fluctuated greatly due to fresh water incursions or typhoon events. Therefore, because the Masan station is directly affected by fresh water, its WT and salinity fluctuations were large. In particular, the salinity level of the Masan station fluctuates exceptionally, as shown by the data from 1 January 2014 to 31 July 2017. The degree of agreement between the three stations for salinity had a low R^2^ (0.09–0.23) in the period. Thus, when the salinity data of the Geojedo and Gadeokdo stations were used to supplement the missing data of the Masan station, these fluctuations were not well revealed (Figure 3c). In addition, the salinity level of the Gadeokdo station showed greater volatility than the level of the Geojedo station because of the effect of fresh water from the Nakdong River.

### 2.3. Temporal Prediction of PSTs Outbreak

**Figure 4:**
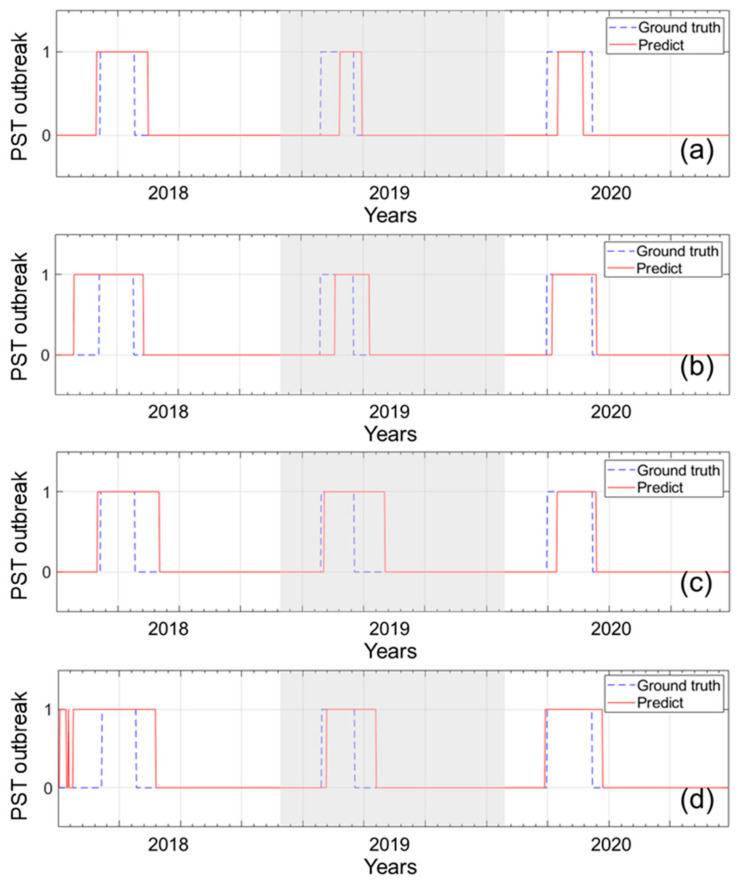
Comparison of ground-truth and the prediction from four LSTM models: (**a**) LSTM-1, (**b**) LSTM-2, (**c**) LSTM-3, and (**d**) LSTM-4. The *x*-axis and *y*-axis represent the time point of test period and the result of PST outbreak, respectively.

To predict the timing of PST outbreaks, we trained and tested LSTM classification models with different combinations of the three environmental factors (WT, tidal height, and salinity). To train and test the models, we used the gap-filled environmental factors of three stations from 2006 to 2017 (4382 days) and from 2018 to 2020 (1096 days), respectively. Table 2 and Figure 4 show the performance results of four LSTM classification models. The compositions of the models have the WT variable in common. The tidal height and salinity factors were included differently for the training models. The LSTM-1 model, which used only the WT variable, had the highest accuracy (0.9). The LSTM-4 model, which used all factors, showed poor performance. In terms of false alarm, i.e., the sum of false negatives and false positives, the LSTM-1 model had the smallest number of 105, and the LSTM-4 model had the greatest value of 154. The LSTM-2 and 3 models had a similar performance, with an accuracy of 0.89. Hence, we chose the LSTM-1 model to predict the timing of PST outbreaks.

## 3. Discussion

### 3.1. Performance of LSTM Models

In this study, two LSTM models were used. LSTM regression and classification models were trained for gap-filling of the environmental data and prediction of PST outbreaks, respectively. The classic proven-useful models have been applied for time-series analysis in various fields [19,20,21,22]. Kim et al. [23] applied auto-regressive integrated moving average (ARIMA), multi-layer perceptron (MLP), and LSTM models to fill the missing period of groundwater levels. ARIMA assume that the present data is a linear function of past data points and past errors [24,25]. They reported that the ARIMA and LSTM models are more accurate than the MLP model. In particular, in the case of the LSTM model, the errors according to the type of input variable was small. In addition, the connection with the before and after data was found to be good when predicting missing values. The error of LSTM model is hardly decreased even if the amount of data increases. Qin et al. [26] used a hybrid model of ARIMA and a deep belief network (DBN) to predict the occurrence of red-tide blooms. ARIMA can express only linear patterns in time series data; however, it is not applicable in nonlinear patterns. With the development of artificial neural network (ANN), machine learning and deep learning approaches become very important nonlinear techniques in the time series forecasting field. Shin et al. [17] proposed a LSTM model for predicting the daily occurrence time series of *Margalefidinium polykrikoides* bloom using satellite-based data. They showed that LSTM model is useful for early prediction of red-tide bloom. Based on these previous literatures, we chose a LSTM model for filling missing values and predicting PST outbreaks.

The LSTM regression model showed good average RMSEs of 0.99 (WT), 24.45 (tidal height), and 1.49 (salinity) for the predicted data at Geojedo station. We used the available LSTM models in Table 1 to fill in missing sections. In the case of tidal height, the model with two stations was available at all stations. On the other hand, the salinity prediction model with two stations was not available at all stations because the salinity data at the Masan station had a higher rate of missing data than the data at the other two stations. In addition, data from the Geojedo station, with the lowest missing data rate, were used most frequently to estimate other factors. Even in the case of tidal height at the Geojedo station, data from other stations were not available; thus, the LSTM was trained with only the tidal height data of this station. The RMSE of the LSTM model showed poor performance, with a value of 58.64. The other two factors of WT and salinity also had poor performances of 7.18 and 2.07, respectively.

To identify the impacts of environmental factors on the performance of the LSTM-based temporal prediction model, we constructed four different combinations of environmental inputs. As a result, the LSTM-1 model trained with only the WT factor showed the best performance, and the LSTM-4 model trained with all factors showed the poorest performance. Moreover, we trained the LSTM models with only tidal height (LSTM-TH) and salinity (LSTM-S) sequence data in three stations. As a result, the accuracy of LSTM-TH and LSTM-S models showed 0.74 and 0.81, respectively. Compared to LSTM-1 model (0.9) with only WT data, the accuracy levels were lower. In the case of recall level, LSTM-TH (0.29) and LSTM-S (0.3) models was very low rather than LSTM-1 model (0.65). This indicates that WT may be the most important factor for predicting the timing of PST outbreaks. We calculated the mean WT value at three stations at the start and the end dates of PST outbreaks during 2018–2020. The mean WT values at the start dates in three years (2018–2020) were 9.83, 11.63, and 11.03, respectively. The mean WT values at the end dates in three years were 15.43, 12.97, and 17.07, respectively. These results suggest that the WT data have a great influence on PST outbreak, but it is difficult to determine the start and end of PST outbreak only with the WT data. To investigate the degree of collinearity between variables, we calculated the condition index by Belsley collinearity diagnostics [27]. This index shows the degree of multicollinearity in a regression design matrix. In the case of three variables of Geoje tidal station, the maximum condition index is 8.4. Kennedy [28] mentioned that the condition index greater than 30 indicates strong collinearity. According to the Kennedy’s report, we concluded that three environmental variables have weak collinearity.

Table 2 shows the performances of the models developed with the gap-filled data of the three tidal stations. Even when the model was trained with only the gap-filled WT of each station, the model showed good performance, with RMSEs of 0.88–0.90. To determine the effectiveness of the gap-filling on temporal prediction of PST outbreaks, we compared LSTM classification models using non-gap filled (LSTM-5) and gap-filled (LSTM-6) data. For training of the LSTM-5 and LSTM-6 models, we used the daily sequence data from 1 January 2014 to 31 June 2017 (1277 days) and from 1 January 2006 to 31 June 2017 (4198 days), respectively. Test data were from 1 February 2018 to 31October 2020 (1003 days). The LSTM-6 model with gap-filled data showed better performance, with an accuracy of 0.9, than the LSTM-5 model with non-gap filled data (accuracy of 0.84). In terms of false alarms, the LSTM-6 model had a low level of 98, whereas the LSTM-5 model had 139. This indicates that gap-filling of data improves the performance of the temporal prediction model for PST outbreaks.

### 3.2. Environmental Factors

**Figure 5:**
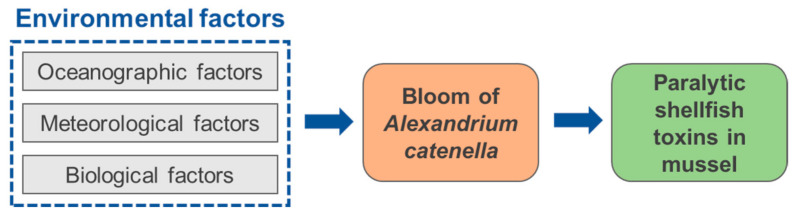
Growth process of PSTs in mussel. Environmental factors include oceanographic, meteorological, and biological factors.

Figure 5 depicts the growth process of PSTs in mussel. *Alexandrium* blooms are caused by various environmental factors, and mussels feed on PST-producing dinoflagellates, including *Alexandrium* species. It would be good to build a direct model to predict PST outbreak through *Alexandrium* blooms; however, it is difficult to collect the relevant daily reports from NIFS and field survey. Due to the lack of the ground truth, we cannot build a good model to predict PST outbreaks through *Alexandrium* bloom. Furthermore, NIFS provides the approximate toxin levels of shellfish. When the PST concentration is greater than the reference value, with 80 μg STX diHCI equivalents 100 g^−1^, they designate the area as prohibited. To generate ground truth data of PST outbreaks, we used the start and the end dates of PST outbreaks provided by NIFS and labelled the value of one in the period of PST outbreaks.

In our study, only WT, tidal height, and salinity, which could be acquired continuously at a daily interval, were used. Our result showed that WT is the most important factor associated with PSTs outbreaks. However, these results are limited to the study area in Korea, and the influence of the environmental factors on PST outbreaks depends on the region. *A. catenella* appears in Osaka Bay, Japan during winter-spring periods. The magnitudes of the abundances and the PST levels are varied depending on years. In this case, the LSTM model trained with only WT might not be appropriate to predict PST outbreaks in Osaka Bay [29,30]. Other oceanographic, biological, and meteorological factors can determine the initiation, development, and decline of *A. catenella* blooms [7,12]. The Tsushima Warm Current creates a counterclockwise current along the coast of Geoje [13]. Such currents make the Geoje coast greatly affected by freshwater discharged from the Nakdong River during torrential rain. Baek et al. [13] found that *A. catenella* blooms occurred during the one or two weeks following the high nitrate + nitrite concentrations and low salinity levels associated with a Nakdong River discharge. High *A. catenella* cell concentrations occurred in March and April when nutrient concentrations were relatively high [15,16]. Their results showed that the plentiful nutrients furnished by freshwater from the Nakdong River enabled the development of *A. catenella* blooms.

In terms of biological factors, Marsden and Shumway [31] mentioned that the first introduction of viable vegetative *A. catenella* cells into the water column could play an important role in development of *A. catenella* blooms. The cells produce resting cysts. This species is widespread in the southern coast of Korea [12]. Thus, the germination of resting cysts leads to the early existence of vegetative cells. The life-cycle transitions of *Alexandrium* species are species-specific and regulated by environmental factors. Eventually, the internal changes of life-cycle stages and the complex external changes in the environment need to be simultaneously to forecast spring blooms most effectively. Kim et al. [32] tracked *A. catenella* from seed-bed to bloom at a hot spot of cyst deposition on the southern coast of Korea from June 2016 to February 2020. They mentioned that cyst germination at a rate of about 73% occurred synchronously in the month of November from 2016 to 2019, when the bottom WT was approximately 15 °C. Overwintering populations initiated growth in March and then proliferated into high-density spring blooms in mid-April 2017, when moderate temperatures (~15 °C) were recorded.

In addition to tidal effects, a major factor in the transport of blooms is wind-induced surface currents. This factor could be a key regulator of cell accumulation along coasts [13,33,34]. Baek et al. [13] found that wind speeds were mostly > 10 ms^−1^ when the population of *A. catenella* gradually expanded around Geojedo and Jinhae Bay. They suggested that strong winds and the surface WT caused physical acceleration of mixing of the entire water column in the spring season. The nutrient loading to the superjacent euphotic layer from bottom layers attributable to this factor and this nutrient supply played an important role in the growth of *A. catenella* population. Moreover, topography and shore geometry can influence cell accumulation of phytoplankton [35]. In conclusion, various coupled factors, including cell dispersal, population accumulation, and bloom distribution, play important roles in the dynamics of *Alexandrium* bloom.

## 4. Conclusions

In this study, we developed a daily temporal prediction model of PSTs in the mussel *M. galloprovincialis* along the Geoje coast and in Jinhae Bay in southern Korean coastal waters using environmental data and an LSTM neural network model. The major results were the following: (i) the timing of PST outbreaks gradually accelerated in a span of three recent years (2018–2020); (ii) as a result of the LSTM regression model for gap-filling of environmental data, the average R^2^ values of WT, tidal height, and salinity over the three stations were 0.92, 0.88, and 0.29, respectively; and (iii) among the four LSTM models, the LSTM-1 model trained with only gap-filled WT sequence data showed the best performance. The LSTM-based temporal prediction model can monitor PST outbreaks. In addition to mussel, NIFS provides information on toxin of various shellfish, such as oyster, manila clam, and scallop. Our study is focused on mussel; however, if toxin information of other shellfish can be collected, the LSTM models developed in this study can be applied. Furthermore, if the LSTM model was expanded to predict PSPs as well as make hourly predictions, it would provide useful data for building a pre-disaster system for PSPs.

## 5. Materials and Methods

### 5.1. Study Area

**Figure 6:**
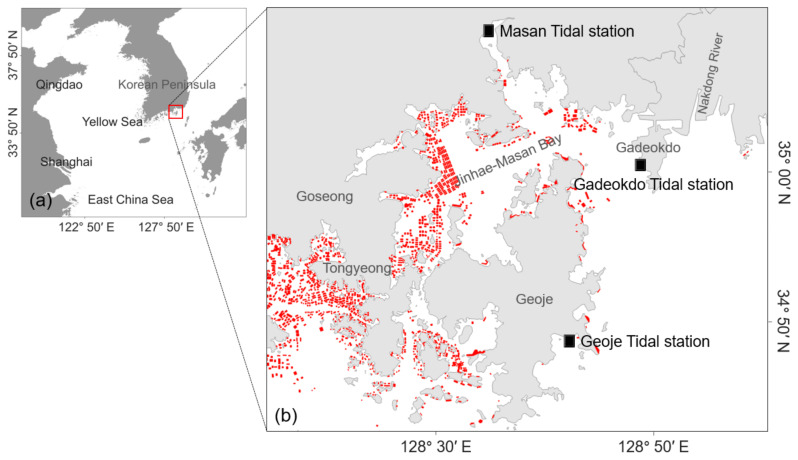
(**a**) Location of the study area on the southern coast in Korean Peninsula. (**b**) Locations of Masan, Gadeokdo, and Geoje tidal stations. The red boxes represent aquaculture farms for shellfish, such as mussel and oyster.

The study area covered the Geoje coast and Jinhae Bay in the southern Korean coastal waters (Figure 6). This area is in nearshore water open toward the outer sea and is affected by the Jeju Warm Current and Tsushima Warm Current. In addition, the area is influenced by tidal effects and fresh water from the Nakdong River located to the northeast [36]. The second largest river in South Korea, the Nakdong River, releases 20% of its discharge during the dry season and 60–70% during the summer monsoon season. *A. catenella* blooms have been occurring along the Geoje coast and in the proximal Jinhae-Masan Bay. Since the first record of a bloom in 1986 [8], outbreaks of PSTs during spring have been reported by the NIFS [11]. In addition, there is considerable shellfish aquaculture along this coast.

### 5.2. Data

**Figure 7:**
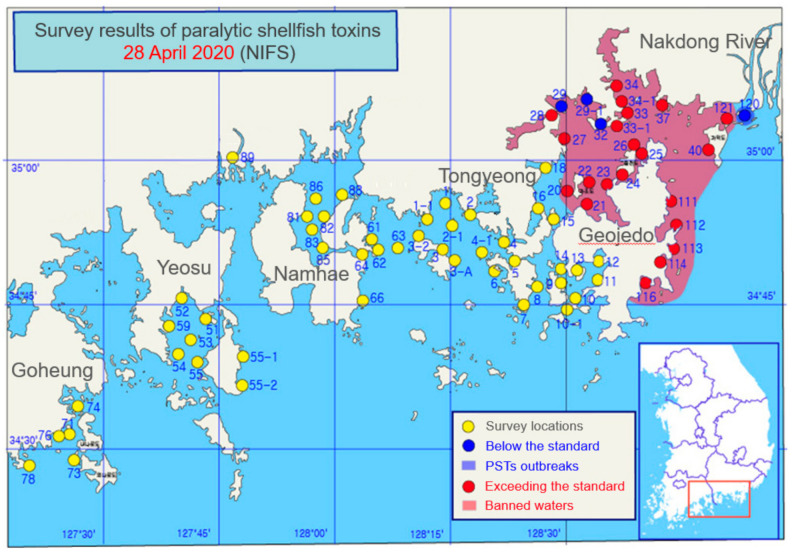
National watch service map at 28 April 2020 provided by NIFS. The map shows spatial occurrence of PSTs and areas where shellfish collection has been banned in the coast.

The start and end dates of PST outbreaks along the Geoje, Gadeokdo, and Masan coasts were provided by the NIFS from 2006 to 2020 [11]. Figure 7 shows an example of National Water Service map for 28 April 2020. There are 129 survey points along the southern coast of the Korean Peninsula, and the PST concentration was measured at each survey point to provide information about whether it is greater than or equal to the reference value (80 μg STX diHCI equivalents 100 g^−^^1^). If the detected PST concentration exceeds the standard value, the area is designated a prohibited area. We generated timing data of 5479 daily PSTs as ground truth data.

Environmental factors data were obtained from the Ocean Data in Grid Framework provided by the Korea Hydrographic and Oceanographic Agency [37]. The agency operates 48 tidal stations along the coast of the Korean Peninsula. The information includes tidal height, WT, salinity, wave height, air temperature, and wind speed. As shown in Table 3, we used the information from the Geojedo, Gadeokdo, and Masan tidal stations, which are located in the study area. Among these three tidal stations, the Gadeokdo station has been in operation for the longest time. We collected WT, tidal height, and salinity data for the 15 years from 2006 to 2020, from three stations, as environmental factor data potentially affecting PST outbreaks. The data were measured at every minute, but we constructed daily data by selecting the first measurement of the day. Finally, a daily data sequence of 5479 data of the three environmental factors was generated for each of three stations.

### 5.3. LSTM Neural Network Model

**Figure 8:**
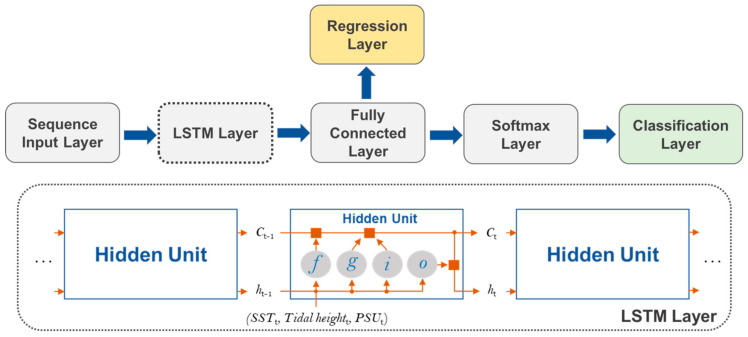
Schemes of LSTM network models for regression and classification tasks, which gap-fill environmental sequence data and temporally predict PSTs outbreak, respectively.

Figure 8 shows the scheme of the LSTM network models. A deep LSTM is a recurrent neural network well-suited to learning the relations of time steps in sequence data [38,39] and model nonlinear functions [40]. In this study, we used two types of LSTM network models for the regression and classification tasks. To fill any day data gaps of environmental factors, a regression output layer was implemented. On the other side, a classification layer was used to predict the timing of PST outbreaks. The input layer feeds WT, tidal height, and salinity sequential daily data into the LSTM model. For regression, the LSTM layer is connected to the fully connected regression layer. On the other side, for classification, the LSTM layer is sequentially connected to the fully connected, softmax, and classification layers. The LSTM layers of both the regression and classification models contain 200 hidden units. Each hidden unit learns the dependencies between previous and current time steps by updating or removing information accumulated from previous hidden units through three control gates, including the input gate (*i*), forget gate (*f*), and output gate (*o*). *C_t_* and *h_t_* represent the cell state and hidden state, which contain information acquired from the previous hidden units and the LSTM output at the current time step (*t*), respectively. *g* indicates a cell candidate, which contains the information to be added.

### 5.4. Performance Assessment

We evaluated the accuracy of the LSTM models for temporal prediction of PST outbreaks in terms of the confusion matrix [41]. The *pst* and *npst* symbols in Table 4 indicate the occurrence timings of PSTs and non-PSTs in the ground truth data, respectively, and the *PST* and *nPST* indicate the occurrence timings of PSTs and non-PSTs in the predicted occurrence of PSTs, respectively. The accuracy ([(1) + (4)]/[(1) + (2) + (3) + (4)]) was evaluated using only the occurrences of PSTs from the ground truth data and the predicted occurrence data.

## Figures and Tables

**Table 1:** RMSEs of LSTM models with test dataset from 1 February 2018 to 31 October 2020. In the case of 2 stations, the environmental factor of the specific station is estimated from the other two stations. For example, water temperature (WT) of Geojedo is estimated from WT values of Gadeokdo and Masan stations.

Variable	Tidal Station	1 Station			2 Stations
		Geojedo	Gadeokdo	Masan	
WT (°C)	Geojedo	-	0.84	1.22	0.89
	Gadeokdo	1.08	-	1.33	0.92
	Masan	2.22	1.82	-	1.91
Tidal height (cm)	Geojedo	-	35.76	19.15	18.45
	Gadeokdo	30.77	-	12.17	10.86
	Masan	33.40	21.09	-	15.53
Salinity (PSU)	Geojedo	-	1.32	1.83	1.33
	Gadeokdo	2.08	-	2.77	2.22
	Masan	3.44	3.69	-	3.45

**Table 2:** Performance of four LSTM classification models for temporal prediction of PSTs outbreak using test dataset.

Models	Factors	(1)	(2)	(3)	(4)	Accuracy
LSTM-1	WT	871	64	41	120	0.90
LSTM-2	WT + Tidal	822	33	90	151	0.89
LSTM-3	WT + Salinity	811	21	101	163	0.89
LSTM-4	WT + Tidal + Salinity	766	8	146	176	0.86

(**1**) True negative; (**2**) false negative; (**3**) false positive; and (**4**) true positive.

**Table 3:** Specification of three tidal stations considered in this study.

Station Name	Latitude (°N)	Longitude (°E)	Availability
Geoje (Ge)	34.80	128.70	1 January 2006–Present
Gadeokdo (Ga)	35.02	128.81	1 January 1977–Present
Masan (Ma)	35.20	128.58	1 December 2002–Present

**Table 4:** Confusion matrix for evaluating the accuracy of the *Sargassum* detection.

		Ground Truth Data	
		False (*npst*)	Ture (*pst*)
The Predicted Result	False (*nPST*)	(1) True negative	(2) False negative
	True (*PST*)	(3) False positive	(4) True positive

## Data Availability

Not Applicable.
